# Long-Term Real-World Effectiveness of Dupilumab vs. Upadacitinib in Early Treatment Responders with Atopic Dermatitis: Results from Central European Health Fund Registry

**DOI:** 10.3390/ijms26094230

**Published:** 2025-04-29

**Authors:** Katarzyna Waligóra-Dziwak, Aleksandra Dańczak-Pazdrowska, Dorota Jenerowicz

**Affiliations:** Department of Dermatology, Poznan University of Medical Sciences, 60-355 Poznan, Poland; aleksandra.danczak-pazdrowska@ump.edu.pl (A.D.-P.); djenerowicz@ump.edu.pl (D.J.)

**Keywords:** atopic dermatitis, real-world effectiveness, dupilumab, upadacitinib

## Abstract

While clinical trials have shown the efficacy of using dupilumab and upadacitinib to treat atopic dermatitis (AD), long-term real-world data remain limited. This retrospective cohort study, utilizing data from the Polish National Health Fund Registry, evaluates the long-term effectiveness of dupilumab versus upadacitinib in early treatment responders, focusing on sustained efficacy outcomes and high levels of skin clearance. Data from the Polish National Health Fund Registry were analyzed for 435 patients; 220 were treated with dupilumab and 215 were treated with upadacitinib, each receiving treatment for at least 40 weeks. Upadacitinib had a faster onset of action, leading to significantly higher rates of complete skin clearance compared to dupilumab at weeks 16 and 28 (EASI100 at week 16: 19.5% vs. 7.3%, *p* < 0.001; week 28: 26.5% vs. 12.7%, *p* < 0.001). Dupilumab showed continuous efficacy gains, ultimately demonstrating superiority in achieving EASI75 and EASI90 at week 40, though no significant difference was observed in achieving EASI100. Both treatments provided sustained dermatological improvement and enhanced quality of life, achieving high levels of skin clearance over the long term.

## 1. Introduction

Atopic dermatitis (AD) is the most prevalent chronic inflammatory skin disorder, affecting up to 20% of individuals throughout their lives [1]. However, the incidence of AD varies significantly depending on geographic region and age group. Recent estimates indicate a global prevalence of 2.6%, affecting approximately 204.05 million individuals worldwide [2]. This includes around 101.27 million adults and 102.78 million children, corresponding to prevalence rates of 2.0% and 4.0%, respectively [2]. The disease is characterized by recurrent episodes of itchy, inflamed, and eczematous skin changes, often following a relapsing–remitting pattern. The condition can develop at any age, though it most commonly manifests in early childhood, with many cases persisting into adulthood [1]. The disease has a strong genetic component [3] and often coexists with other atopic conditions [4]. This genetic predisposition can interact with environmental factors to trigger and exacerbate the disease. Various mechanisms contribute to its development and symptoms. A weakened epidermal barrier, often occurring due to a filaggrin deficiency [5], can induce inflammation and allow T-cell infiltration. The immune response in AD is primarily driven by T helper 2 cells, further compromising the skin barrier. Additional factors influencing AD include imbalances in skin microbiota, systemic immune dysregulation, and neuroinflammation, which plays an important role in itching [6,7].

AD pathogenesis involves multiple inflammatory cytokines, contributing to disease progression by promoting inflammation; these include interleukin 4 (IL-4), interleukin 13 (IL-13), interleukin 22 (IL-22), interleukin 31 (IL-31), thymic stromal lymphopoietin (TSLP), and interferon-gamma (IFN-γ) [8,9], which transduce signals through the Janus kinase–signal transducer and activator of transcription (JAK-STAT) signaling pathway, playing a critical function in regulating the immune response central to AD [10,11]. Currently, we are witnessing the development of advanced therapies targeting these pathways, including both biologic drugs as well as JAK inhibitors, with dupilumab and upadacitinib being notable examples.

Dupilumab is a fully human monoclonal antibody, administered via injection. It is approved for treating moderate and severe AD [12]. It is the first biological drug approved for this condition, representing a breakthrough in targeted AD therapy. It works by blocking the shared α subunit of the IL-4 and IL-13 receptors, effectively disrupting the signaling pathways of IL-4 and IL-13 [12], both of which are pivotal in mediating the inflammatory responses associated with AD. Its safety and efficacy profile has been well-established through numerous clinical trials [13,14,15].

Upadacitinib represents a significant member of the JAK inhibitor class, selectively targeting JAK1 to block the signaling of various proinflammatory mediators, such as IL-4, IL-13, IL-22, IL-31, TSLP, and IFN-γ [16,17,18]. The rapid and maintained efficacy of upadacitinib in the treatment of AD has been demonstrated in previous studies, reinforcing its role as a valuable systemic (orally administered) therapeutic option [16,17,18].

Currently, there are few studies assessing the long-term efficacy of dupilumab versus upadacitinib in adults as well as adolescents with AD. So far, the available research has primarily been restricted to a limited number of phase 3 and 4 clinical trials, with observation periods of no more than 24 weeks (wks). Specifically, we refer to the 24-week Heads Up study [19] and 16-week analysis from the Level Up study [20]. Real-world data are also limited—only three observational studies compared the two medications, but their observation periods were no longer than 24 wks, and sample sizes were generally modest [21,22,23].

In this retrospective cohort study utilizing data from the Polish National Health Fund Registry, we aim to evaluate the long-term efficacy of dupilumab versus upadacitinib in early treatment responders. Our analysis focuses on long-term outcomes and the achievement of high levels of skin clearance, which we regard as clinically significant due to their substantial impact on reducing the effects of AD on psychological well-being, sleep, as well as overall life quality.

The assessment of treatment efficacy in clinical trials can be influenced by multiple methodological factors that may not fully reflect real-world clinical practice. These factors include, among others, controlled access to a prescribed amount of emollient, the standardized use of specific topical corticosteroids, and predefined dosing regimens that may not account for patient-specific adjustments. While clinical trials provide rigorous, high-quality evidence, their controlled environments may not capture the full variability of patient adherence, lifestyle influences, or socioeconomic barriers that impact treatment outcomes in real-world settings. This underscores the importance of real-world data, which complement clinical trial findings by offering meaningful insights into the actual therapeutic effectiveness of treatments across diverse patient populations, helping to identify real-world usage patterns, thereby bridging the gap between clinical research and everyday medical practice.

To our knowledge, this is the first study to provide results on the long-term effectiveness of dupilumab versus upadacitinib in early treatment responders with AD, with a treatment duration of up to 40 wks in a real-world setting. While previous studies have primarily focused on short-term outcomes from randomized controlled trials, our research offers evidence on sustained treatment responses in a nationally representative cohort, filling a gap in the current literature regarding head-to-head effectiveness over extended treatment periods but also providing insights into treatment durability and comparative response dynamics among patients who initially respond to therapy.

## 2. Results

### 2.1. Demographics and Patient Baseline Characteristics

The analysis included a total of 435 patients, comprising both adults (*n* = 329; aged 18 to ≤75 years) and adolescents (*n* = 106; aged ≥ 12–17 years), who received either upadacitinib or dupilumab. Among them, 215 were treated with upadacitinib (105 female [48.8%]; mean age of 28.2 years; SD: 14.0), and 220 were treated with dupilumab (102 female [46.4%]; mean age of 29.3 years; SD: 13.9). The demographic and baseline characteristics were generally comparable between groups (Table 1). Key baseline disease activity measures included a mean [SD] Eczema Area and Severity Index (EASI) score of 32.2 [11.1] for the dupilumab group and 30.1 [10.3] for the upadacitinib group, while mean [SD] Dermatology Life Quality Index (DLQI) scores were 20.4 [5.6] and 20.3 [6.0], respectively (Table 1).

### 2.2. Efficacy Outcomes

**Figure 1:**
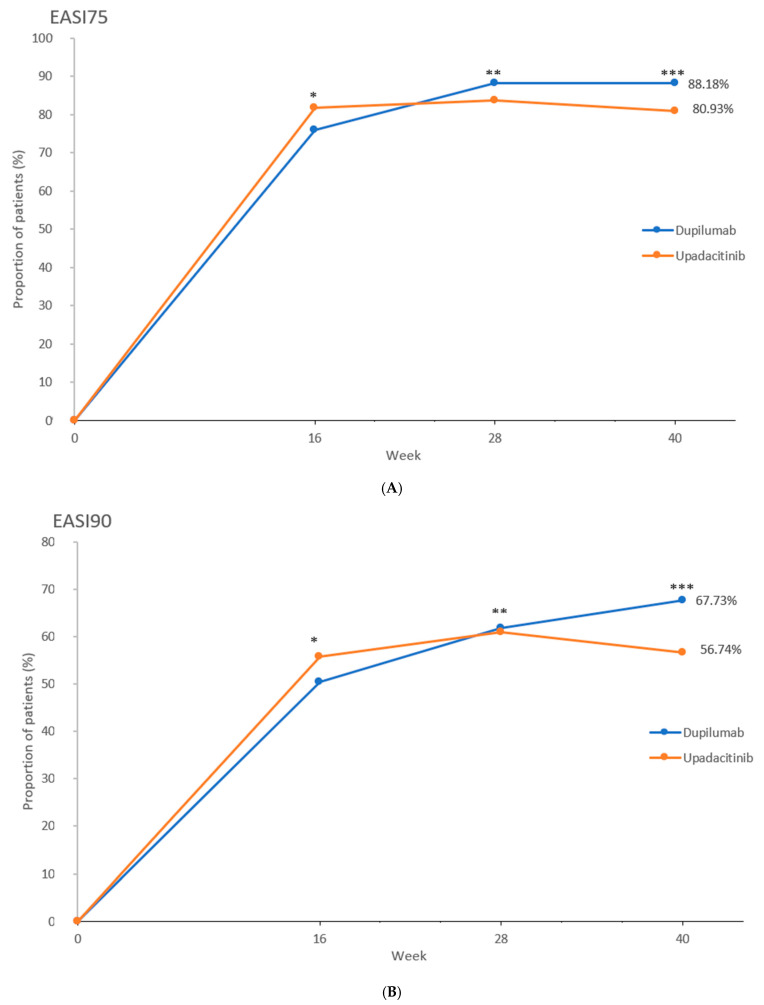

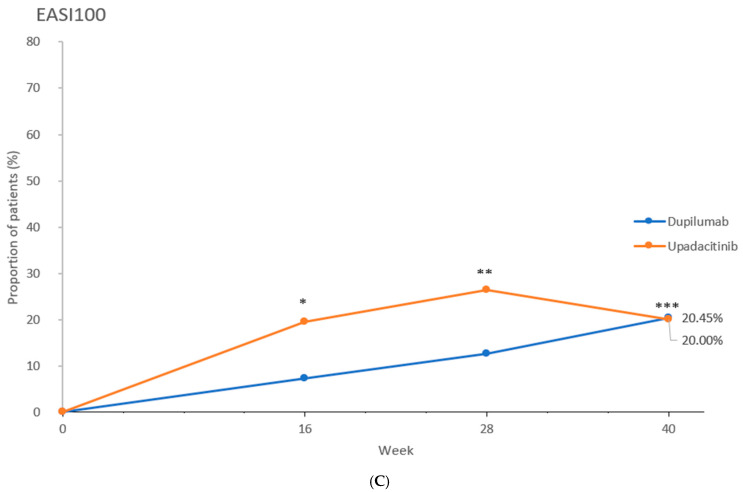
The proportion of patients achieving (**A**) at least 75% improvement in the Eczema Area and Severity Index (EASI75) at weeks 16, 28, and 40 (* *p* = 0.161; DUPI 95% CI [70.3–81.6]; UPA 95% CI [76.7–87.0]; ** *p* = 0.230; DUPI 95% CI [83.9–92.4]; UPA 95% CI [78.8–88.7]; *** *p* = 0.0498; DUPI 95% CI [83.9–92.4]; UPA 95% CI [75.7–86.2]) (**B**) at least 90% improvement (EASI90) at weeks 16, 28, and 40 (* *p* = 0.306; DUPI 95% CI [43.8–57.1]; UPA 95% CI [49.2–62.5]; ** *p* = 0.927; DUPI 95% CI [55.4–68.2]; UPA 95% CI [54.4–67.5]; *** *p* = 0.024; DUPI 95% CI [61.5–73.9]; UPA 95% CI [50.1–63.4]) (**C**) 100% improvement (EASI100) at weeks 16, 28, and 40 in the dupilumab and upadacitinib groups; (* *p* < 0.001; DUPI 95% CI [3.8–10.7]; UPA 95% CI [14.2–24.8]; ** *p* < 0.001; DUPI 95% CI [8.3–17.1]; UPA 95% CI [20.6–32.4]; *** *p* = 1.000; DUPI 95% CI [15.1–25.8]; UPA 95% CI [14.7–25.3]). Data are presented as percentages of patients achieving the respective endpoints at each time point. Statistical comparisons between treatment groups were performed using Pearson’s chi-square (χ^2^). Abbreviations: CI, confidence interval; DUPI, dupilumab; UPA, upadacitinib.

**Figure 2:**
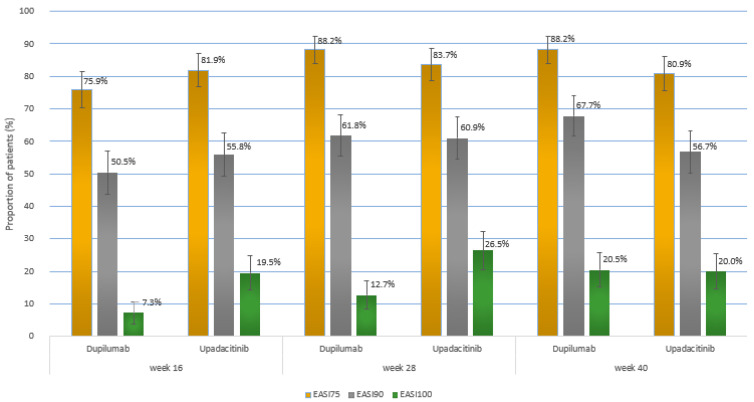
The proportion of patients achieving at least 75% improvement in the Eczema Area and Severity Index (EASI75), at least 90% improvement (EASI90), and 100% improvement (EASI100) at weeks 16, 28, and 40 in the dupilumab and upadacitinib groups. Data are presented as percentages of patients achieving the respective endpoints at each time point. Error bars represent 95% confidence intervals.

For the primary efficacy outcome, the proportion of patients achieving EASI75 at wk 40 was 88.2% (*n* = 194) in the dupilumab group and 80.9% (*n* = 174) in the upadacitinib group (*p* = 0.0498) (Figure 1A and Figure 2; Table 2).

**Figure 3:**
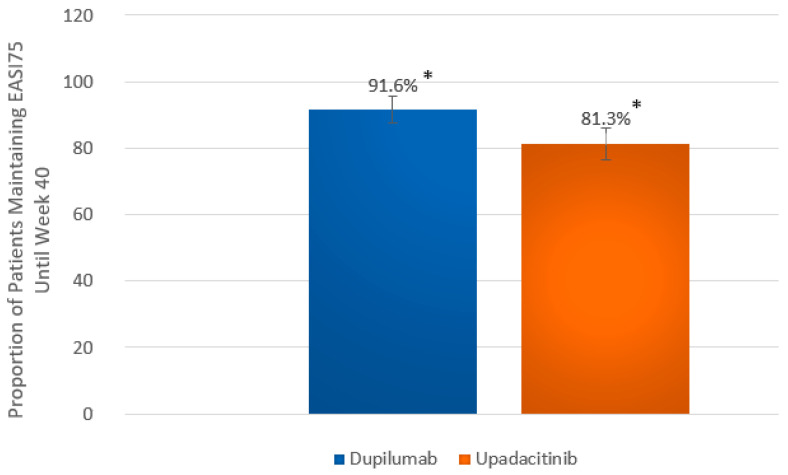
The proportion of patients maintaining at least 75% improvement in the Eczema Area and Severity Index (EASI75) from week 16 to week 40 in the dupilumab and upadacitinib groups. Data are presented as the percentage of patients who sustained their response over the 24-week follow-up period. Statistical comparisons between treatment groups were performed using Pearson’s chi-square (χ^2^). * *p* = 0.008; error bars represent 95% confidence intervals.

**Figure 4:**
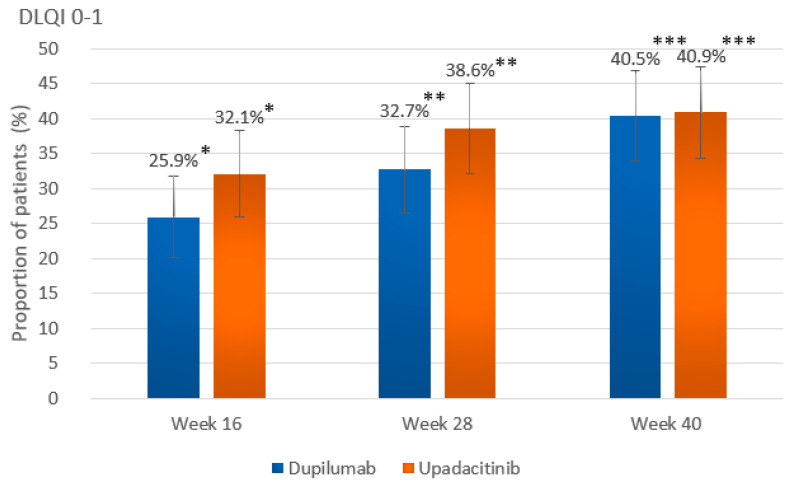
The proportion of patients experiencing little to no impact of the disease on quality of life, defined as a Dermatology Life Quality Index (DLQI) score of 0–1, at weeks 16, 28, and 40 in the dupilumab and upadacitinib groups. Data are presented as the percentage of patients achieving DLQI 0–1 at each time point. Statistical comparisons between treatment groups were performed using Pearson’s chi-square (χ^2^). * *p* = 0.188; ** *p* = 0.238; *** *p* = 0.997; error bars represent 95% confidence intervals.

When key secondary outcomes at wk 40 are considered, a significantly greater proportion of patients in the dupilumab group versus the upadacitinib group achieved almost complete skin clearance, expressed as EASI90 (149 [67.7%] vs. 122 [56.7%]; *p* = 0.024) (Figure 1B). Complete skin clearance at wk 40, expressed as EASI100, was achieved in 20.5% (*n* = 45) of dupilumab-treated individuals and 20.0% (*n* = 43) of upadacitinib-treated individuals, with no significant difference (*p* = 1.000) (Figure 1C). The proportion of patients maintaining EASI75 from wk 16 until wk 40 was significantly higher in the dupilumab group than in the upadacitinib group (153 out of 167 who achieved EASI75 at wk 16 [91.6%] versus 143 out of 176 who achieved EASI75 at wk 16 [81.3%]; *p* = 0.008) (Figure 3). Proportions of patients showing little to no effect of the disease on quality of life, expressed as DLQI 0-1 at wk 40, were comparable between groups (40.5% vs. 40.9%), with no statistical difference (*p* = 0.997) (Figure 4).

The onset of action was faster for upadacitinib and led to the achievement of EASI100 in a significantly greater proportion of upadacitinib-treated individuals compared to the dupilumab-treated patients at wk 16 and 28 (EASI100 at wk 16: [19.5%] vs. [7.3%]; *p* < 0.001; EASI100 at wk 28: [26.5%] vs. [12.7%]; *p* < 0.001) (Figure 1; Figure 2; Table 2). EASI75 at wk 16 was achieved by 81.9% (*n* = 176) of upadacitinib-treated patients versus 75.9% (*n* = 167) of dupilumab-treated patients, while EASI90 at wk 16 was obtained by 55.8% (*n* = 120) of upadacitinib patients versus 50.5% (*n* = 111) of dupilumab patients. At wk 16, numerically higher proportions of individuals treated with upadacitinib achieved all evaluated EASI improvement outcomes (EASI75, EASI90, and EASI100); however, statistical significance was exclusively shown for the achievement of EASI100 (Supplementary Table S1). The proportion of patients showing complete or almost complete improvement in quality of life (DLQI 0-1) at wk 16 was numerically higher for upadacitinib compared to dupilumab treatment (69 [32.1%] vs. 57 [25.9%]; *p* = 0.188).

**Figure 5:**
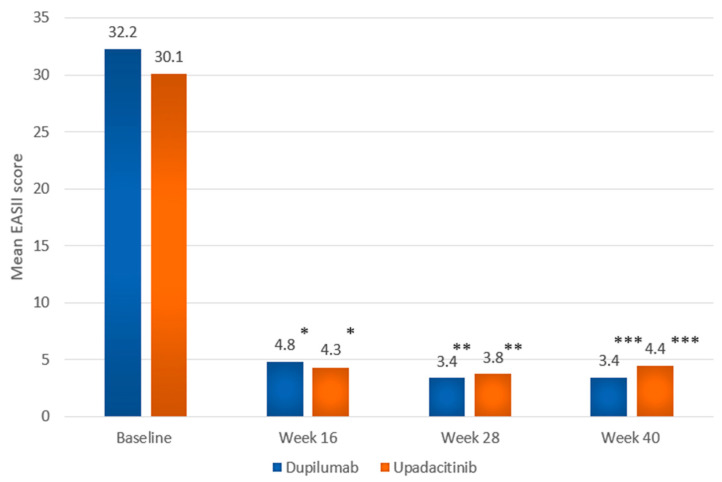
Mean EASI score at baseline, and at weeks 16, 28, and 40 in the dupilumab and upadacitinib groups. Data are presented as mean values. Statistical comparisons between treatment groups at each time point were performed using the Mann–Whitney U test. * *p* = 0.039; ** *p* = 0.234; *** *p* = 0.077.

**Figure 6:**
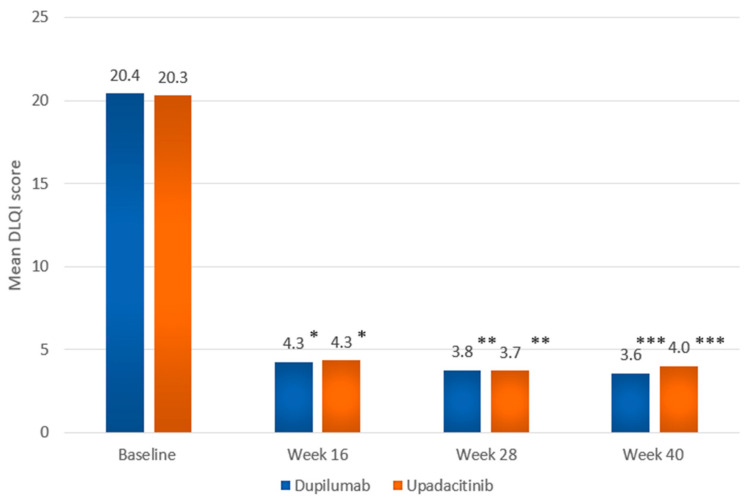
Mean DLQI score at baseline, and at weeks 16, 28, and 40 in the dupilumab and upadacitinib groups. Data are presented as mean values. Statistical comparisons between treatment groups at each time point were performed using the Mann–Whitney U test. * *p* = 0.464; ** *p* = 0.204; *** *p* = 0.857.

At wk 28, a numerically higher proportion of dupilumab-treated individuals compared to those treated with upadacitinib achieved EASI75 (194 [88.2%] vs. 180 [83.7%]) as well as EASI90 (136 [61.8%] vs. 131 [60.9%]); the differences were not statistically significant (Supplementary Table S1). As mentioned earlier, EASI100 was attained at wk 28 by a significantly greater proportion of individuals in the upadacitinib group. The proportion of individuals showing high levels of improvement in quality of life (DLQI 0-1) at wk 28 was numerically higher in the upadacitinib group than in the dupilumab group (83 [38.6%] vs. 72 [32.7%]; *p* = 0.238). Mean values of EASI and DLQI showed a marked decrease compared to baseline in both groups at consecutive follow-up points, starting with the first evaluation visit at wk 16 (Figure 5 and Figure 6, Supplementary Table S1).

## 3. Discussion

Long-term results, as well as the achievement of high levels of skin clearance, are important in the context of the persistent nature of AD and its influence on patients’ quality of life [24]. AD is a recurrent and chronic condition requiring effective long-term medication; thus, both a rapid response [25] and the maintenance of high treatment efficacy over prolonged periods is essential. Moreover, a shift in AD treatment goals toward the complete removal of residual skin lesions is currently being observed, as in psoriasis, where higher improvement scores such as PASI90 or PASI100 are important indicators of treatment effectiveness for systemic therapies [26].

To the best of our knowledge, to date, results are only available from three real-world studies comparing dupilumab and upadacitinib, with two of them being 12-week studies [21,22] and one being a 24-week study (analyzing data from 23 patients) [23]. The scarcity of high-quality, real-world data comparing these two treatments is concerning, particularly as there is growing interest in understanding how targeted systemic therapies like dupilumab and upadacitinib perform in routine clinical practice. Besides the limited real-world data, results from two clinical trials directly comparing dupilumab and upadacitinib in the treatment of AD are currently available (24-week Heads Up study, 16-week Level Up study with an extension period of up to 32 wks) [19,20].

At wk 16, in the Heads Up study (upadacitinib 30 mg vs. dupilumab as per its label, with patients who received rescue topical/systemic immunomodulatory treatment considered nonresponders for binary endpoints [19]), EASI75 was attained by 72.4% of patients receiving upadacitinib and 62.6% receiving dupilumab (*p*  =  0.007). The achievement of EASI100 at wk 16 was reported in more patients in the upadacitinib group (28.4% vs. 7.9%; *p*  <  0.001) [19]. Additionally, at wk 16, a significantly greater proportion of upadacitinib-treated patients attained EASI90 compared with the dupilumab group (61.6% vs. 40.3%; *p*  <  0.001) [19].

At wk 16 of the ongoing Level Up study, which evaluated the effects of upadacitinib (15 mg or 30 mg) with a flexible dosing regimen based on clinical response vs. dupilumab as per its approved label (with patients requiring topical rescue treatment considered nonresponders [20]), upadacitinib-treated patients demonstrated significantly higher rates of extensive skin clearance (EASI90, [40.8%] vs. [22.5%], *p* < 0.001; EASI100, [14.8%] vs. [5.6%]; *p* < 0.001) [20]. The study extension to 32 wks for those who did not achieve EASI75 at wk 16 has not yet yielded results.

At wk 24, in the Heads Up study, which is currently the longest study directly comparing dupilumab and upadacitinib with available results, numerically higher proportions of patients treated with upadacitinib attained all evaluated EASI improvement outcomes (EASI75, EASI90, and EASI100); however, as in the present study at wk 28, significance was shown exclusively for the achievement of complete skin clearance (EASI100, [27.8%] vs. [13.6%]; *p*  <  0.001; EASI90, [56.6%] vs. [49.5%]; *p*  =  0.07; EASI75 [65.3%] vs. [61.2%]; *p*  =  0.29) for upadacitinib versus dupilumab (without adjustment for multiplicity) [19].

In the present study, we investigated the long-term effectiveness of dupilumab vs. upadacitinib in early treatment responders with AD. The results demonstrated the faster onset of action and the capacity to attain higher levels of dermatological improvement with upadacitinib compared to dupilumab, particularly after the first 16 wks of observation. Over time, the differences diminished, with dupilumab showing stable improvement in efficacy through wk 40 and demonstrating superiority in achieving the primary efficacy outcome. Upadacitinib-treated individuals attained significantly higher rates of EASI100 at wk 16 and 28; however, at wk 40, there was no significant difference in EASI100 achievement between the two patient groups.

These findings align with the outcomes of the placebo-controlled, long-term phase 3 studies (AD UP—examining upadacitinib vs. placebo, LIBERTY AD CHRONOS—examining dupilumab vs. placebo; no direct comparisons between the two medications), where greater response rates were observed at wk 16 with upadacitinib in the AD UP study (especially at the higher 30 mg dose) compared to dupilumab (in CHRONOS); however, this difference gradually decreased over time [27]. Silverberg et al. suggested that, in clinical practice, wk 16 may be too early to fully assess the complete therapeutic benefits of biologics [27]. This is because, in the initial stages of treatment, a larger proportion of patients met the stricter efficacy criteria of EASI90 and IGA 0/1 with JAK inhibitors than with biologics; however, over the long-term maintenance phase, effectiveness appeared largely comparable for the majority of patients [27].

Specifically, the AD UP study (upadacitinib 15 mg or 30 mg plus steroids applied topically vs. placebo plus steroids applied topically) [17,28], which closely reflected real-world therapeutic approaches for AD patients, demonstrated that EASI75 was achieved at wk 16 by 64.3% and 76.9% of patients in the 15 mg and 30 mg groups, respectively. By wk 52, EASI75 was achieved by 50.8% of patients in the 15 mg group and by 69.0% in the 30 mg group. At wk 16, EASI90 was achieved by 42.8% and 63.3% of patients in the 15 mg and 30 mg groups, respectively, while at wk 52, the proportions were 37.7% and 55.4%. EASI100 was achieved by 11.7% and 22.6% of patients at wk 16 and by 13.1% and 23.6% at wk 52 in the 15 mg and 30 mg groups, respectively [28]. In the LIBERTY AD CHRONOS study (dupilumab 300 mg every two wks plus steroids applied topically) [14], the proportion of patients that achieved EASI75 was 69% at wk 16 and 65% at wk 52. EASI90 was achieved by 40% of patients at wk 16 and 51% at wk 52. No EASI100 data from the LIBERTY AD CHRONOS study were identified [14,29].

However, since higher rates of long-term upadacitinib response have also been reported (Measure Up 1, Measure Up 2; upadacitinib 15 mg or 30 mg/day vs. placebo, topical medications for AD allowed from wk 16) [16], it is important to recognize that outside of the context of direct head-to-head trials, drawing comparisons between different studies is inherently challenging. Variations in analytical approaches, study designs, and protocols for rescue treatment—such as the timing of its allowance—introduce complexities that limit the reliability of cross-trial evaluations [27]. Additional factors, including differences in dosing regimens, the duration of washout periods for prior topical and systemic therapies, and disparities in inclusion and exclusion criteria (such as patient age, disease severity, and responder classification), further complicate data interpretation. Inconsistencies in eligibility criteria across trials may lead to challenges in drawing meaningful comparisons. Moreover, the length of the washout period can significantly influence early trial outcomes [27]. All of this highlights the unique significance of head-to-head studies while giving no less importance to real-world comparisons, which reflect actual treatment patterns in clinical practice.

In the present study, the higher performance rates of dupilumab compared to upadacitinib at wk 40 could have been influenced by several factors. These include the more common use of the lower 15 mg dose of upadacitinib, the lack of control over the effects of concomitant topical or systemic medications (as this information was not captured in the registry), and the absence of a washout period. Additionally, the selection of patients who were early responders at wk 16 may have influenced the results. Nevertheless, the findings provide valuable insights into real-world usage patterns and the effectiveness of both medications, particularly in relation to the duration of performance outside of controlled clinical trials.

## 4. Materials and Methods

### 4.1. Study Design

A retrospective cohort study utilizing data from the Polish National Health Fund Registry was conducted to compare the efficacy of dupilumab versus upadacitinib in individuals with AD, who received treatment for at least 40 wks.

The study analyzed data from 220 patients treated with dupilumab, who began treatment after 1 November 2022, and were the first to reach 40 wks of therapy. It also analyzed 215 patients treated with upadacitinib, who started treatment after 1 November 2022, and were the first to reach 40 wks of therapy by 1 December 2024, at the latest. Data concerning efficacy were gathered at baseline as well as at 3 consecutive control points: week 16 (wk 16), week 28 (wk 28), and week 40 (wk 40). An elasticity of +/− 14 days for scheduling follow-up visits was allowed in case of unforeseen events.

As reporting data to the National Health Fund Registry is mandatory, the analysis included data from all Polish medical centers providing treatment with dupilumab or upadacitinib under the Polish National Health Fund Program.

The data reported in the study were provided by and with the agreement of Polish National Health Fund Registry authorities. This study was approved by the appropriate Institutional Research Ethics Committee and conducted in accordance with the Declaration of Helsinki and applicable regulations.

### 4.2. Patients

The patients whose data were analyzed included adults (ranging from 18 to 75 years) and adolescents (ranging from 12 to 17 years), all diagnosed with AD and treated with upadacitinib or dupilumab under the Polish National Health Fund Program. Patients who qualified for treatment had a disease severity defined by a baseline EASI score of ≥20. Additionally, they were candidates for systemic treatment, documenting an inadequate response to the use of topical emollients and corticosteroids. Individuals aged 12–17 years had to meet at least one of the following criteria: the previous failure of systemic immunosuppressive therapy, contraindications to systemic immunosuppressive therapy that prevented its use, or adverse effects that made the continuation of systemic immunosuppressive therapy impossible. Individuals aged 18 and older had to meet one of the following criteria: the previous failure of cyclosporine treatment, contraindications to cyclosporine that prevented its use, or adverse effects that made the continuation of cyclosporine treatment impossible. Adequate organ function was determined based on blood laboratory test results, in accordance with the current *Summary of Product Characteristics* (SmPC). Patients were eligible for inclusion in the absence of significant comorbidities that constituted a contraindication to therapy, as determined by the attending physician, and after the exclusion of pregnancy or breastfeeding.

Exclusion criteria for continued participation in the program included an inadequate response to treatment (assessed at specified follow-up visits), defined by the fulfillment of both of the following criteria: failure to achieve at least a 50% reduction in the Eczema Area and Severity Index (EASI50) score, and failure to achieve an improvement in quality of life, as assessed by DLQI or Children’s Dermatology Life Quality Index (CDLQI), of at least four points compared to baseline values. Other reasons for treatment discontinuation included the occurrence of diseases or conditions that, in the opinion of the attending physician, prevented further continuation of treatment. This included among others, hypersensitivity reactions to any active substance or excipient; toxicity requiring treatment discontinuation; or a lack of cooperation and the patient’s non-compliance with medical recommendations. Patients did not bear the cost of the treatment.

The analysis included early treatment responders, defined as patients who achieved EASI50 or an improvement in quality of life, as assessed by the DLQI/CDLQI scale, of at least four points compared to baseline values at wk 16 and who continued treatment until wk 40. The analyzed group consisted of 435 patients, whose data were fully available in the registry.

### 4.3. Treatment Dosage and Concomitant Medications

The analyzed data considered patients receiving dupilumab, given as a subcutaneous injection—300 mg every two wks (q2w) in adults, or 200 mg/300 mg q2w in adolescents (˂60 kg or ≥60 kg, respectively)—following a loading dose of 600 mg in adults and 400 mg/600 mg in adolescents, or upadacitinib, administered orally once daily as a 15 mg or 30 mg extended-release tablet for at least 40 wks.

**Figure 7:**
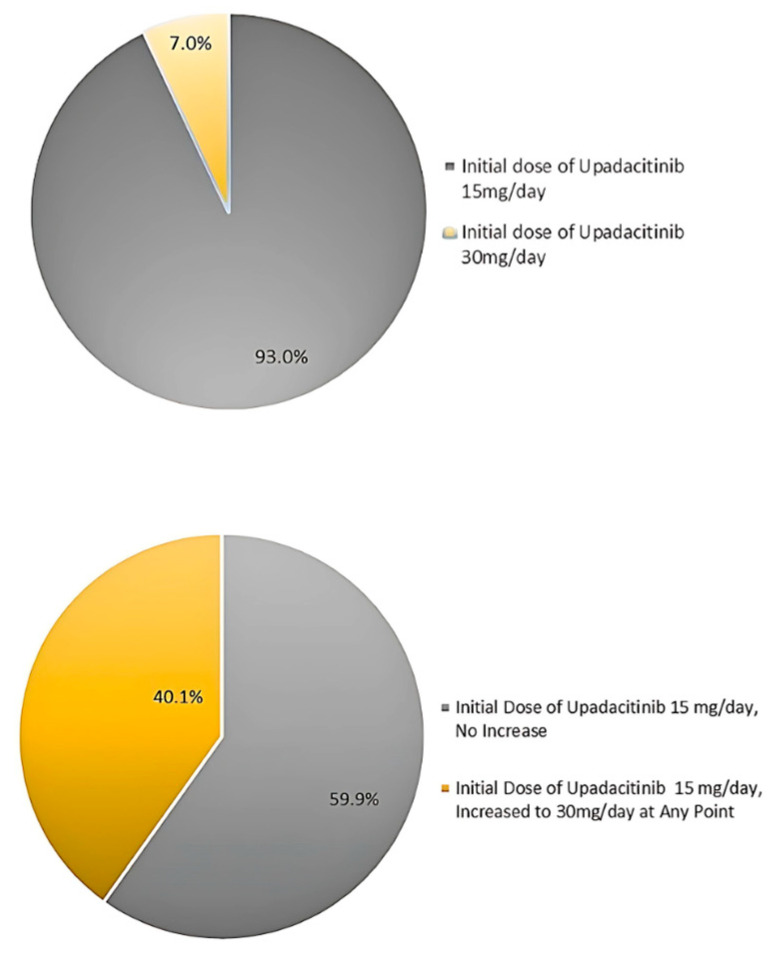
Upadacitinib dosage (15 mg/day or 30 mg/day) throughout treatment period. Data available for patients treated with upadacitinib with full-term observation (365 days, *n* = 185).

The upadacitinib dosage was initially selected and adjusted during the treatment at the physician’s discretion. Data available for patients treated with upadacitinib with full-term observation (365 days, *n* = 185) showed that 93.0% (*n* = 172) of patients initially received a 15 mg dose of upadacitinib, while 7.0% (*n* = 13) received an initial dose of 30 mg/day. The percentage of patients whose initial dose was 15 mg/day and who had at least one administration of the drug at a dose of 30 mg/day in their treatment history was 40.1% (*n* = 69), compared to the 59.9% (*n* = 103) whose treatment was not escalated (Figure 7).

Previous treatment with another JAK inhibitor or dupilumab within the Polish National Health Fund Program occurred in the upadacitinib group in fewer than five patients (for each medication, see Table 1). No patients in the dupilumab group had previously been treated with any other medication under the Polish National Health Fund Program (upadacitinib, baricitinib, tralokinumab, or abrocitinib).

Restrictions were not imposed on the prior use of systemic treatments for AD. Patients were not required to undergo washout periods before starting the treatment. This approach allowed for the inclusion of patients who had previously been treated with systemic therapies, providing a more representative sample of the broader AD patient population. Additionally, the use of concomitant treatments, whether topical or systemic, was permitted throughout the treatment period, at the physician’s discretion.

### 4.4. Efficacy Parameters

Since long-term outcomes, such as sustained improvements in skin condition, and the achievement of high levels of skin clearance were parameters of interest, we focused on evaluating how well the treatments performed in achieving these goals over extended periods. The primary efficacy outcome was defined as the achievement of EASI75 at wk 40. Key secondary efficacy outcomes were defined as the proportion of patients maintaining EASI75 from wk 16 until wk 40; the achievement of EASI90 at wk 40; the achievement of EASI100 at wk 16, wk 28, and wk 40; and the proportion of patients achieving DLQI 0-1 at wk 40.

### 4.5. Statistical Analysis

Statistical analysis was performed using R software (version 4.1.2). As reporting efficacy data at specific follow-up points to the National Health Fund Registry is strictly mandatory, and as the study specifically included patients who continued treatment until wk 40 (which required obligatory assessments at three follow-up visits at wk 16, 28, and 40), the data in the registry were complete. The analysis encompassed both descriptive and inferential objectives. Categorical variables were presented as counts and percentages, while continuous variables were expressed as means with standard deviations (SD). Comparisons between the two groups were conducted using the Mann–Whitney U test for continuous variables and Pearson’s chi-square (χ^2^) test for categorical variables, with statistical significance set at *p* < 0.05. Where relevant, 95% confidence intervals (CIs) were also calculated. All of the above data and statistical analyses were provided by the National Health Fund. Multiplicity-adjusted outcomes were subsequently derived through a hierarchical testing method (fixed sequence procedure).

## 5. Limitations

This study had some limitations. As in the Level Up study, the results were presented based on data from all upadacitinib doses combined, without stratification between the 15 mg and 30 mg doses. This approach was undertaken due to the flexible dosing regimen, which was tailored to the patient’s clinical condition according to the physician’s judgment, allowing adjustments in dosage over short periods as needed.

Although not necessarily a limitation, as treatment with a specific drug is typically prescribed on a long-term basis to patients who show at least satisfactory improvement after an initial treatment period, this study focused on the long-term effects in early treatment responders (patients attaining EASI50 at wk 16 or adequate DLQI/CDLQI improvement). Some of the higher response rates observed in this study may be partly attributed to the inclusion of early treatment responders. It would be beneficial to conduct another long-term study of patients who did not initially respond to treatment.

The use of topical medications, as well as systemic immunosuppressants, was permitted during the treatment period; however, this information was not captured or specified in the registry. The absence of detailed documentation regarding the use of these concurrent treatments may have potentially influenced the results, leading to higher response rates compared to previous long-term phase 3 studies. Nonetheless, the data reflect real-world clinical practice, where the use of concomitant medications is typically unrestricted. As such, the findings also provide valuable insights into the effectiveness and usage patterns of the analyzed treatments in a real-world setting, closely mirroring typical treatment approaches in routine care.

There were also limitations related to the design of the registry itself, which did not include data on factors such as race, ethnicity, body mass index, patient-reported outcomes other than the DLQI/CDLQI, or side effects. The absence of these variables restricts the ability to assess the broader impact of analyzed treatments fully. In addition, future research could analyze treatment effectiveness by sex and age group, provided that larger sample sizes are available.

To our knowledge, this is the largest real-world study analyzing the efficacy of dupilumab versus upadacitinib, with the highest number of patients included and the longest duration of observation. However, the sample size was limited by the requirement of completing 40 wks of treatment under the Polish National Health Fund Program. Nevertheless, the study achieved statistically significant primary outcome, and most key secondary outcomes were also significant.

## 6. Conclusions

Overall, both dupilumab and upadacitinib demonstrated sustained efficacy, achieving high levels of skin clearance and improvement in quality of life over the long term. Upadacitinib exhibited a faster response, with higher initial efficacy rates, especially in terms of achieving stringent skin improvement thresholds. Dupilumab showed continuous gains in efficacy, ultimately surpassing upadacitinib in terms of achieving the primary outcome at wk 40. Both treatments proved effective for AD patients.

Our data may offer insights for future treatment strategies; however, further studies with larger patient populations are needed to confirm these findings for a better understanding of the long-term impact and role of both therapies in AD management.

As treatment options for AD continue to expand, the need for direct comparisons between available therapies is becoming increasingly important. While numerous clinical trials have demonstrated the efficacy and safety of individual treatments, there remains a scarcity of long-term comparative studies assessing their effectiveness over extended periods.

Given the current breakthrough in access to targeted therapies for AD, including biologics and JAK inhibitors, equipping medical practitioners with high-quality, evidence-based data is of the utmost importance. Reliable comparative studies can help clinicians navigate the growing therapeutic landscape, tailor treatments to individual patient needs, and ensure informed decision-making. Additionally, long-term data on efficacy, safety, and patient-reported outcomes will be crucial in shaping treatment guidelines and improving the overall standard of care for individuals living with AD.

Greater numbers of long-term, head-to-head studies with primary endpoints set at extended timeframes, along with comprehensive real-world data on AD treatments, are essential for providing clinicians with the necessary evidence to thoroughly assess the durability and effectiveness of systemic therapies for AD. By facilitating a deeper understanding of long-term treatment outcomes, these data would support more precise, patient-centered treatment decisions for individuals with moderate-to-severe AD, ultimately optimizing therapeutic strategies.

## Figures and Tables

**Table 1:** Patient demographics and clinical characteristics at baseline.

Characteristic	Patients, No. (%) Dupilumab, 300 mg q2w (Adults) Dupilumab 200/300 mg q2w (Adolescents; <60 kg or ≥60 kg, espectively) (*n* = 220)	Upadacitinib, 15 mg/30 mg Dose at Physician’s Discretion (*n* = 215)
Sex		
Male	118 (53.6)	110 (51.2)
Female	102 (46.4)	105 (48.8)
Age, mean (SD) [range], yr	29.3 (13.9) [12–75]	28.2 (14.0) [12–75]
Age category		
12 to < 18	47 (21.4)	59 (27.4)
18 to < 40	119 (54.1)	107 (49.8)
40 to <65	50 (22.7)	46 (21.4)
65 to ≤75	4 (1.8)	3 (1.4)
EASI, mean (SD) [range]	32.2 (11.1) [20.0–71.5]	30.1 (10.3) [20.0–65.4]
DLQI, mean (SD) [range]	20.4 (5.6) [8–30]	20.3 (6.0) [5–30]
Previous treatment in the Polish National Health Fund Program		
- with upadacitinib	0	0
- with baricitinib	0	<5
- with abrocitinib	0	0
- with tralokinumab	0	0
- with dupilumab	0	<5

Abbreviations: EASI, Eczema Area and Severity Index; DLQI, Dermatology Life Quality Index; SD, standard deviation; q2w, every two weeks.

**Table 2:** Primary and key secondary efficacy outcomes.

Outcome	Dupilumab Group 300 mg q2w (Adults) 200/300 mg q2w (Adolescents; <60 kg or ≥60 kg, Respectively) (*n* = 220)	Upadacitinib Group 15 mg/30 mg Dose at Physician’s Discretion (*n* = 215)	*p* Value
Primary outcome			
EASI75 at wk 40 ^a^	194 (88.2) [83.9–92.4]	174 (80.9) [75.7–86.2]	*p* = 0.0498
Key secondary outcomes			
Proportion of patients maintaining EASI75 from wk 16 until wk 40 ^a^	153 (91.6) ^b^ [87.4–95.8]	143 (81.3) ^c^ [75.5–87.0]	*p* = 0.008
EASI90 at wk 40 ^a^	149 (67.7) [61.5–73.9]	122 (56.7) [50.1–63.4]	*p* = 0.024
EASI100 at wk 16 ^a^	16 (7.3) [3.8–10.7]	42 (19.5) [14.2–24.8]	*p* < 0.001
EASI100 at wk 28 ^a^	28 (12.7) [8.3–17.1]	57 (26.5) [20.6–32.4]	*p* < 0.001
EASI100 at wk 40 ^a^	45 (20.5) [15.1–25.8]	43 (20.0) [14.7–25.3]	*p* = 1.000
DLQI 0-1 at wk 40 ^a^	89 (40.5) [34.0–46.9]	88 (40.9) [34.4–47.5]	*p* = 0.997

Abbreviations: EASI, Eczema Area and Severity Index; DLQI, Dermatology Life Quality Index; q2w, every two weeks; wk, week. ^a^ No. (%) [95% CI]. ^b^ percentage out of 167 patients who achieved EASI75 at week 16. ^c^ percentage out of 176 patients who achieved EASI75 at week 16.

## Data Availability

The original contributions presented in this study are included in the article and Supplementary Material. Further inquiries can be directed to the corresponding author.

## Supplementary Materials

The following supporting information can be downloaded at: https://www.mdpi.com/article/10.3390/ijms26094230/s1.

## Abbreviations

The following abbreviations are used in this manuscript:

AD	Atopic Dermatitis
CDLQI	Children’s Dermatology Life Quality Index
CI	confidence interval
DLQI	Dermatology Life Quality Index
DUPI	dupilumab
EASI	Eczema Area and Severity Index
EASI75	at least 75% improvement in the Eczema Area and Severity Index
EASI90	at least 90% improvement in the Eczema Area and Severity Index
EASI100	100% improvement in the Eczema Area and Severity Index
IFN-γ	interferon gamma
IL-4	interleukin 4
IL-13	interleukin 13
IL-22	interleukin 22
IL-31	interleukin 31
JAK-STAT	Janus kinase–signal transducer and activator of transcription signaling pathway
q2w	every two weeks
SD	standard deviation
SmPC	*Summary of Product Characteristics*
TSLP	thymic stromal lymphopoietin
UPA	upadacitinib
wk	week
wks	weeks
