# Supplementary material for: Long-Term Real-World Effectiveness of Dupilumab vs. Upadacitinib in Early Treatment Responders with Atopic Dermatitis: Results from Central European Health Fund Registry
Source: Int J Mol Sci. 2025 Apr 29;26(9):4230. doi: 10.3390/ijms26094230 (PMC12072912; doi:10.3390/ijms26094230)
Supplement: Supplementary file 1 [file ijms-26-04230-s001.zip › Supplementary Table 1.pdf]

Table S1. Other secondary efficacy outcomes.

Outcome	Dupilumab group 300mg q2w (adults) 200/300 mg q2w (adolescents; <60 kg or ≥60 kg, respectively) (n=220)	Upadacitinib group 15mg/30mg Dose at physician's discretion (n=215)	P value*
**EASI75 at wk 28** [a]	194 (88.2) [83.9 – 92.4]	180 (83.7) [78.8 – 88.7]	P = 0.230
**EASI90 at wk 28**[a]	136 (61.8) [55.4 – 68.2]	131 (60.9) [54.4 – 67.5]	P = 0.927
**EASI75 at wk 16**[a]	167 (75.9) [70.3 – 81.6]	176 (81.9) [76.7 – 87.0]	P = 0.161
**EASI90 at wk 16**[a]	111 (50.5) [43.8 – 57.1]	120 (55.8) [49.2 – 62.5]	P = 0.306
**EASI at wk 40, mean (SD) [range]**	3.4 (5.0) [0-33.7]	4.4 (6.1) [0-44.7]	P = 0.077
**EASI at wk 28, mean (SD) [range]**	3.4 (3.9) [0-22.4]	3.8 (5.4) [0-41.8]	P = 0.234
**EASI at wk 16, mean (SD) [range]**	4.8 (4.9] [0-22.0]	4.3 (5.2) [0-22.6]	P = 0.039
**DLQI at wk 40, mean (SD) [range]**	3.6 (4.1) [0-22]	4.0 (5.1) [0-27]	P = 0.857
**DLQI at wk 28, mean (SD), [range]**	3.8 (3.7) [0-18]	3.7 (4.6) [0-28]	P = 0.204
**DLQI at wk 16, mean (SD), [range]**	4.3 (3.9) [0-19]	4.3 (4.5) [0-22]	P = 0.464
**DLQI 0-1 at wk 28**[a]	72 (32.7) [26.5 – 38.9]	83 (38.6) [32.1 – 45.1]	P = 0.238
**DLQI 0-1 at wk 16**[a]	57 (25.9) [20.1 – 31.7]	69 (32.1) [25.9 – 38.3]	P = 0.188

Abbreviations: EASI, Eczema Area and Severity Index; DLQI, Dermatology Life Quality Index; SD, standard deviation; q2w, every two weeks; wk, week. [a]No. (%) [95% CI]. * without adjustment for multiplicity
